# Paracetamol and Ibuprofen in the Treatment of Fever and Acute Mild–Moderate Pain in Children: Italian Experts’ Consensus Statements

**DOI:** 10.3390/children8100873

**Published:** 2021-09-30

**Authors:** Mattia Doria, Domenico Careddu, Raffaele Iorio, Alberto Verrotti, Elena Chiappini, Giulio Michele Barbero, Flavia Ceschin, Laura Dell’Era, Valentina Fabiano, Michele Mencacci, Francesco Carlomagno, Maria Libranti, Teresa Mazzone, Antonio Vitale

**Affiliations:** 1 Italian Pediatricians Federation (FIMP), 30015 Chioggia, Italy; mattia.doria@gmail.com; 2 Italian Pediatricians Federation (FIMP), 28100 Novara, Italy; drcareddupediatra@gmail.com; 3 Department of Translational Medical Science, University of Naples Federico II, 80131 Naples, Italy; riorio@unina.it; 4 Department of Pediatrics, University of Perugia, 06100 Perugia, Italy; ver.albert@yahoo.it (A.V.); antoniovitale1@libero.it (A.V.); 5 Division of Pediatric Infectious Diseases Infective Diseases, Anna Meyer Children’s University Hospital, 50100 Florence, Italy; 6 Department of Health Sciences, University of Florence, 50100 Florence, Italy; 7 Independent Researcher, 12100 Cuneo, Italy; barbero.giulio@libero.it; 8 Italian Pediatricians Federation (FIMP), 33170 Pordenone, Italy; ceschinf.ped@gmail.com; 9 Fondazione IRCCS Ca’ Granda, Ospedale Maggiore Policlinico, 20100 Milan, Italy; laura.dellera@policlinico.mi.it; 10 Department of Pediatrics, Vittore Buzzi Children’s Hospital, University of Milan, 20100 Milan, Italy; valentina.fabiano@unimi.it; 11 Independent Researcher, 06100 Perugia, Italy; michele.mencacci@gmail.com; 12 Independent Researcher, 80100 Naples, Italy; francescocarlomagno1959@gmail.com; 13 Italian Pediatricians Federation (FIMP), 95100 Catania, Italy; marialibranti@libero.it; 14 Independent Researcher, 00100 Rome, Italy; mazzoneteresa14@gmail.com

**Keywords:** fever, pain, children, primary care, hospital, emergency department

## Abstract

Fever and pain are challenging symptoms in children and adolescents and are common reasons for consultations in primary care and hospital. Paracetamol and ibuprofen are currently the only recommended drugs for treating fever in Italy, but the therapeutic approaches are discrepant in the different settings. In Italy, paracetamol and ibuprofen are the most prescribed analgesics for acute mild–moderate pain in children; however, their use is often inappropriate in that fever is over-treated and pain is under-treated. An Italian board of experts analyzed the motivations for the misalignment between daily practice and guidelines of fever and acute mild–moderate pain management of the territory and hospitals. The expert opinion consensus process underscored the appropriate use of paracetamol and ibuprofen according to clinical scenarios, patients’ profiles, and the safety features of the drugs. Although patients’ profiles can indicate different benefits from paracetamol or ibuprofen, critical issues of fever and acute mild–moderate pain management persist in primary care and hospitals. These expert opinion consensus statements can be an across-the-board tool to harmonize the routine practice between the territory and hospitals, especially under special conditions (at-risk for dehydration, coagulation disorder patients, etc.). It can also promote educational activity about fever and acute mild–moderate pain management to enhance the milestones already achieved by Italian pediatricians.

## 1. Introduction

Fever and pain are challenging symptoms in children and adolescents [1,2,3] and are common reasons for consultations in primary care and hospital admissions. The therapeutic approaches are discrepant with the evidence-based recommendations in the various settings [4,5,6,7]. The management of fever is characterized by overtreatment, often owing to “fever phobia” [8], whereas pain is undertreated [7], leading to untimely and inadequate analgesia.

In Italy, there are incorrect dosages and low adherence to the above-mentioned guidelines by healthcare professionals and caregivers, and these practices can be dangerous for the health of children [6,8], with regional differences that may mirror the varied backgrounds of pediatricians. In the absence of a clear and univocal definition of discomfort [1], the prevalent approach to febrile patients still focuses on lowering the temperature by antipyretics [8].

Pain management is still suboptimal and uneven, especially in emergency departments (EDs), with inadequate evaluation and treatment [7,9,10]. The approach to pain in primary care is heterogeneous [4], yet pain management requires systematic approaches [11]. Although paracetamol and ibuprofen are the most prescribed analgesics for acute mild–moderate pain in children, their use is inappropriate in most pediatric cases in Italian EDs [10,12].

For the treatment of febrile children, paracetamol and ibuprofen are currently the only recommended drugs in Italy [13]; paracetamol is indicated since birth, whereas ibuprofen is indicated starting from three months of age [13,14].

This document provides insights about the proper use of paracetamol and ibuprofen for the treatment of fever and mild–moderate pain in children in real life of clinical settings (the territory, EDs, and pediatric departments) in Italy. Discussing the evidence and clinical guidelines, these consensus statements, based on expert opinion, are meant to define across-the-board practices for fever and acute mild–moderate pain in children and to align the approaches among the different settings.

## 2. Materials and Methods

The present consensus document is focused on:

(a) The current real-life management of pediatric patients with fever and acute mild–moderate pain in the hospital (ED and pediatric department) and territory settings (gaps, needs, and best practices);
(b) The hallmarks of paracetamol and ibuprofen for children with fever and acute mild–moderate pain (efficacy, contraindications, and the appropriateness of use);
(c) Different categories of patients requiring paracetamol or ibuprofen as appropriate treatments.

### 2.1. Clinical Scenarios

We analyzed three clinical scenarios: fever, acute mild–moderate pain (trauma, headache, otitis, pharyngitis, etc.), and fever and mild–moderate pain in specific patient profiles (dehydration, comorbidities, etc.). Analysis of the three clinical scenarios was divided according to three healthcare settings: primary care (territory), EDs, and (pediatric) hospital departments.

### 2.2. The Experts

Three panels of Italian pediatricians were involved: A national panel that constituted the steering committee and two macroregional (central–northern and central–southern Italy) panels. Five pediatricians constituted the national board, five the central–northern panel, and four the central–southern panel.

The experts of the national board were included based on their institutional affiliations, and those of the two macroregional panels on clinical experience in pediatric hospitals and primary care. All of the experts were involved also according to their publications in the field. The selected experts participated in the entire consensus process.

The project groups (Hippocrates Sintech and Aristea International) provided scientific–methodological and organizational assistance, guiding the experts throughout the consensus process by emails and phone calls, drafting web meeting minutes, and ensuring the timely development of the project.

### 2.3. Literature Search

A literature review provided a framework to promote the discussion among the experts involved. The used PICO criteria were:

- Population: children (age: 0–18 years);
- Intervention: medical management;
- Comparator: paracetamol versus ibuprofen;
- Outcomes: subjective and objective;
- Setting: outpatients and inpatients.

The literature search was performed using the PubMed database and the items were selected according to the following criteria: The years of 2015–2021; children aged birth to 18 years; study types involving clinical trials, prospective or retrospective cohort observational studies, case-control and cross-sectional studies, systematic reviews and meta-analyses, and guidelines. We excluded non-English language publications, case reports, letters, editorials, and grey literature.

The Boolean terms for retrieving citations were:

- Fever AND children NOT coronavirus NOT cancer (11,196 items);
- Acute mild–moderate pain AND children NOT coronavirus NOT cancer NOT anesthesia (12 items);
- Fever AND acute mild-moderate pain AND children NOT coronavirus NOT cancer (2 items);
- Oral ibuprofen AND acute mild–moderate pain AND children NOT coronavirus NOT cancer (2 items);
- Paracetamol AND obese children (32 items);
- Ibuprofen AND obese children (6 items);
- Pain assessment AND disabled children (73 items).

The screening for pertinence according to titles, abstracts, and full texts yielded 31 items.

### 2.4. Procedure

This expert opinion consensus process aims to define statements that enable increasing the certainty of clinical decisions [15]. In this study, we defined a statement as a clinical benefit of paracetamol or ibuprofen for the management of fever and acute mild–moderate pain in children in the territory, EDs, and pediatric departments. We applied a modified Delphi method, which is a qualitative, participative, and comparative tool by administering questionnaires and face-to-face meetings [16]. This method encompassed three phases: an explorative phase to identify experts to involve, objectives, and topics; an analytic phase to collect data and expert opinion to rank the statements that we then evaluated through consecutive rounds; an evaluative phase to assessing the data collected through questionnaires and online or in-person meetings.

### 2.5. Design

Expert opinions were gathered through specific techniques facilitating group discussion, as described below (workshop).

**Figure 1:**
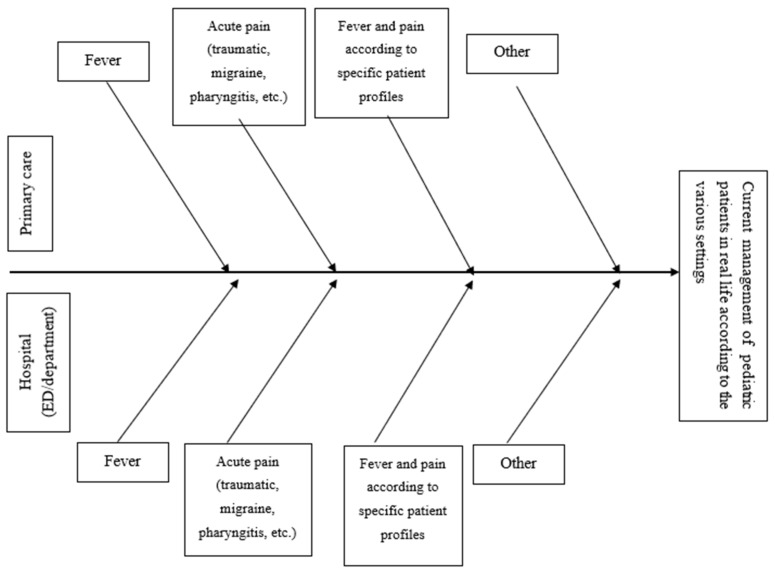
Current management of pediatric patients in real life according to the various settings. ED, emergency department.

In Workshop 1, we applied the Ishikawa or fishbone diagram, which is also known as the “cause–effect” diagram [17] and is suitable to synthesize the brainstorming results of small expert groups. This tool allowed to explore the current management of pediatric patients with fever and acute mild–moderate pain in different real-life settings according to needs, mistakes, and best practices. The analyzed parameters were fever, acute mild–moderate pain, and fever plus mild–moderate pain. Figure 1 displays the Ishikawa diagram used for the current consensus statements.

In Workshop 2, we applied a simplified version of the SWOT analysis, adopting only strengths and weaknesses, and highlighted the expert point of view about the clinical benefits and contraindications of paracetamol and ibuprofen. Moreover, according to the different settings and specific patient profiles, we pointed out their proper use.

The results achieved in Workshops 1 and 2 formed the basis for formulating the statements.

### 2.6. Voting

Each statement was rated by a unipolar five-point Likert scale (Strongly disagree = 1; Disagree = 2; Undecided = 3; Agree = 4; Strongly Agree = 5), with an agreement level cut-off of 80% (convergent answers on 4 and 5 points).

### 2.7. Workflow Events

During the first national web meeting, held on 16 June 2020, the following issues were presented and shared: methods and scientific rationale, topics to appraise, settings to analyze, statements to process, and level of agreement to achieve. The Ishikawa diagram (Workshop 1) and a modified SWOT analysis (Workshop 2) were applied, and the statements were delivered. During the second web meeting, held on 16 July 2020, and the third web meeting, held on 17 September 2020, Workshops 1 (Ishikawa diagram) and 2 (modified SWOT analysis) were repeated (macroregional point of view), and the statements were discussed and completed with voting by the two macroregional panels. Table 1 summarizes the steps of Workshops 1 and 2.

During the fourth national web meeting (20 October 2020), the global results of the workshops and statement voting were presented, and each statement was verified and refined.

## 3. Results

Expert opinions were collected through group discussions with the workshops described in the method. The results achieved in Workshops 1 and 2 formed the basis for formulating the statements.

### 3.1. Workshop 1 (Ishikawa Diagram) Outcomes

In Workshop 1, the results of the analysis of the discussions of the three panels outlined the pattern of fever and acute mild–moderate pain management in primary care and hospital settings (Table 2). The emerged widespread critical issues underscored the need for a consensus statement document.

### 3.2. Fever in Primary Care Settings

Communication was acknowledged as a priority, since it is often insufficient, underused, or heterogeneous. Information is essential for the family, but also for healthcare providers (HCPs) such as pediatricians, nurses, and pharmacists. This priority is due to the different views among HCPs in the various settings in the same territory that may disorient parents. Fever phobia—recently amplified during the COVID-19 pandemic—is detectable in parents and HCPs who manifest hyper-sensibility or limited tolerance to fever.

It was suggested to enhance communication, especially for parents, taking advantage of social media and using coherent messages in simple language, especially regarding general recommendations about the use of drugs and specific guidelines for the administration of paracetamol and ibuprofen. Educational activities should be extended to schools and television programs.

The experts underlined the paramount importance of the continuous education of all HCPs and suggested that pediatricians should train other HCPs in favoring homogeneity and agreement. It is essential to align the messages between hospital and territory settings. Educational activities should be geared toward each territory and macro-area, with a uniform approach within the same administrative area (city, region, or local healthcare administration). The central–southern panel underlined the importance of spreading coherent educational messages based on simple concepts (no physical tools and no preventive administration of drugs). These messages should be shared between territory and hospital settings.

Currently, the antipyretic dose is calculated according to either the age or the weight range. Instead, the dose must be recommended according to the weight of each child. The dosage of paracetamol for infants, even according to the Italian guidelines, is not univocal.

The opinion of the expert panel was that the alternated or combined use of ibuprofen and paracetamol is often improper. The administration routes are critical and regard the personalization of the dose; yet, a distinction between drops, syrup, and suspension is lacking.

The opinion of the expert panel was that a misconception about a superior efficacy and manageability of the rectal route has been spread among parents. The rectal route has limitations in administering the correct dose, given the variable gut absorption [18]. The opinion of the expert panel was that parents perceive ibuprofen as more effective than paracetamol. This perception may be due to the use of ibuprofen at maximum dosage compared to paracetamol, which is often underdosed, especially when administered through the rectal route. Consequently, the rectal route should never be the first-line choice but prescribed only in the presence of vomiting. Moreover, this misconception can explain the increased use of ibuprofen as a first-line treatment in the territory and EDs.

Fever prevention during convulsive crisis is not in compliance with the guidelines; thus, HCPs detect shortcomings in the management of these patients.

The critical issues linked to caregivers are self-medication, self-management of drugs, tendency to observe the degree of temperature as being more than the general status, pursuing the lowering of the temperature, and lacking knowledge of the different side effects of drugs (ibuprofen has different side effects).

### 3.3. Acute Mild–Moderate Pain in Primary Care Settings

The loco-regional difference in managing acute mild–moderate pain in children may be ascribable to the different backgrounds of the pediatricians. We should consider management in terms of pain assessment and treatment, since the appraisal of pain is as valuable as the efficacy of the treatment. The real-life experience of the expert panel is that the systemic aspect of pain is under-evaluated in all Italian regions—only topical therapy is often adopted. The use of scales for pain assessment and re-assessment is incorrect or lacking. The software and computerized medical records used in hospitals generally do not have sections for measuring pain. However, insisting the use of pain scales is paramount because the current methods are based on empirical and indirect measurements is.

The management of pain treatment is often inadequate or missing for prophylaxis, as is the repeated treatments during the day without considering the entire daily dose and in the planned treatment of continuous acute mild–moderate pain (otitis, pharyngitis, etc.). The on-demand approach is frequently used instead of the planned use of drugs.

Similar to fever, the experts underlined the need for reinforcing educational activities through meetings and training with other HCPs (pediatricians, orthopedics, otolaryngologists, etc.) that can increase the awareness of pain in children. In particular, the objective is to increase orthopedics’ attitudes toward evaluating and treating pain in children. Since pain scales are not routinely used, the expert panel recommended that use of scales with strong evidence be more promoted in the primary care setting [19]. Particular attention should be paid to the management of disabled children since they are not able to express their pain.

### 3.4. Fever and Acute Mild–Moderate Pain for Specific Patient Profiles in Primary Care Settings

The risk of dehydration in the management of fever and pain in children in primary care is not always considered, and improper use of ibuprofen in children with Kawasaki’s disease is still detectable, as well as in patients with pneumonia and varicella (Kawasaki’s disease is not a common diagnosis and can be detected after several days of a fever).

The risk of hemorrhage is not considered in at-risk patients (a lack of awareness of pediatricians about the risk of hemorrhage with NSAIDs).

Moreover, caution is required for subjects with acute viral or bacterial infections, underlying chronic pathologies (co-morbidities), infants, gastrointestinal diseases, hemorrhage, and risk of dehydration and acute gastroenteritis.

In children with several pathologies, self-prescription by the families must be opposed and the therapy must be decided and prescribed by doctors.

### 3.5. Fever and/or Acute Mild–Moderate Pain in General and in Specific Patient Profiles in Hospital Care Settings

Children with fever or pain are among those patients who most frequently access Eds and can present different levels of complexity. The critical issues in hospital are the same as those found in the primary care setting. Additional critical issues in hospital are the improper use of paracetamol for fever by the intravenous injection route rather than by oral administration and the alternating use of paracetamol and ibuprofen. More attention on comorbidities and other pathologies is required. Not all EDs are specialized in pediatrics which can explain this picture and can lead to the inadequate management of pediatric patients.

The reference hospital center should highlight, sensitize, and share the risk factors of each child with all HCPs, e.g., writing the risk factors on the dismissal letter. Children with chronic and complex diseases deserve special attention.

Training about prophylaxis for predictable pain, such as that related to procedures, is needed even for ED-based HCPs.

It is essential to also sensitize parents to pain and its best management. In particular, the central–southern panel highlighted the excessive use of self-prescribed ibuprofen by the family for fever in children with dehydration and gastroenteritis, possibly explained by an over-prescription of this drug by pediatricians.

### 3.6. Workshop 2 (Modified SWOT Analysis) Outcomes

Fever and/or acute mild–moderate pain in general and in specific patient profiles

- The dosage and administration of paracetamol are easily achieved;
- The side effects of ibuprofen are also correlated to specific categories of at-risk children (e.g., patients with hemorrhagic susceptibility);
- The main advantage of paracetamol versus ibuprofen is its possible administration even in the first days of life and every 6 h;
- The efficacy, costs, and side effects ratio makes paracetamol a first-line fever treatment, mainly for reducing of discomfort;
- In patients with dehydration, the safety of paracetamol is superior to ibuprofen;
- The antalgic efficacy of paracetamol and ibuprofen for acute mild–moderate pain is similar;
- Parents have misconceptions about the superior efficacy and manageability of the rectal route. The rectal route has limitations in terms of administering the correct dose, given the variable gut absorption. The expert opinion was that parents perceive ibuprofen as more effective than paracetamol. The perception and satisfaction of parents and patients in real life may be due to the use of ibuprofen at the maximum dosage compared to paracetamol, which is often underdosed, especially when administered through the rectal route [20] (referring not to the bench mark, but to the perception and satisfaction of parents and patients in the real life). Consequently, the rectal route should never be the first-line choice but prescribed only in the presence of vomiting;
- The opinion of the experts was that the risk of ibuprofen is higher not only in patients with pneumonia, but also with infectious diseases;
- The opinion of the experts was that in patients with varicella, the use of paracetamol is safer than ibuprofen;
- The risk of ibuprofen, as a first-line treatment, is related to the presence of bacterial and primitive diseases, such as tumors, whereby pain and inflammation are epiphenomena. The anti-inflammatory effect of ibuprofen could hide the real cause of the pain linked to pathologies such as arthritis;
- Ibuprofen must be used with caution in children with nephropathy [21].

Specific comments of the central–northern and central–southern panels emerged during Workshop 2 are reported in Table 3.

During the consensus process, the assessments of the expert panels discussed evidence-based recommendations and their personal experience.

The national board elaborated on four expert opinion statements that were rated by the two macroregional (central–northern and central–southern) panels (Table 4). These statements refer to fever and acute mild–moderate pain in the three clinical scenarios (primary care, EDs, and hospital departments).

The Italian guidelines show that the efficacy of ibuprofen is not superior to that of paracetamol, and highlight their similar safety profiles, yet the types of adverse events are different. Therefore, in light of precision medicine, it is possible to identify the patient categories for whom the two drugs are most appropriate.

### 3.7. Statements

**Statement** **1** . Recommendations for the use of paracetamol and ibuprofen in primary care and emergency settings should overlap (* agreement necessary regardless of the setting).

- Infants require particular attention in terms of fever management;
- Fever should be distinguished from pain;
- It would be helpful to underline certain concepts: not alternating ibuprofen and paracetamol therapy; the guidelines are not consistent everywhere.

According to a recent systematic review, although combined or alternating therapy reduces the temperature more effectively than monotherapy, the benefit on child discomfort was not clinically meaningful [22]. This evidence cannot bolster the combined or alternating use of the two drugs compared to monotherapies, in agreement with the majority of international recommendations [13,22,23].

**Statement** **2.** The guidelines suggest that the efficacies of paracetamol and ibuprofen are comparable (* in terms of efficacy, a 15 mg/kg dose of paracetamol overlaps a 10 mg/kg dose of ibuprofen).

- There is no difference in superiority in terms of efficacy between paracetamol and ibuprofen [13,14];
- The safety profiles of the two drugs are similar but differ according to the type of reported adverse events for the treatment of fever and pain in children. Given the widespread use and the evidence, paracetamol and ibuprofen are associated with rare and specific side effects at the recommended doses [24,25].

**Statement** **3.** Paracetamol showcases a good safety profile when used at the recommended dose of 15 mg/kg 4 times/day maximum (* not to overstep the daily dose of 60 mg/kg; the route of administration and the age of the child are important, e.g., in neonates and infants, the dose should be adjusted to 12.5 mg/kg every 6 h if given by the IV route)

- The dosing of paracetamol must be established according to body weight;
- The maximum recommended dose of paracetamol is safe and cautious. For pain, the toxicity threshold dose of paracetamol (single dose) can be 120 mg/kg [26].

For febrile children, the AGREE II method appraises the guidelines of the Italian Society of Pediatrics, as those with the highest-quality score in terms of methodology, applicability, and transparency [1].

Compared to ibuprofen, at the recommended doses, the liver toxicity of paracetamol is more predictable and liver injury occurs when a dose of 80–100 mg/kg/die is exceeded. At the recommended doses, paracetamol can be safer than NSAIDs for patients with advanced liver disease, but dose adjustments are advised [27]. A dose of 120–150 mg/kg is a potentially toxic dose. A dose of 60 mg/kg/day is the recommended therapeutic dose in children over three months of age or weighing more than 7 kg [13,26,28].

**Statement** **4** . The use of paracetamol is more appropriate in specific conditions: Children at risk of dehydration or dehydrated children and children with varicella, pneumonia, Kawasaki’s disease, and coagulation disorders (* dehydration is a frequent condition in infants with fever).

- In patients with hepatic impairment, paracetamol is recommended for fever and pain management;
- Dehydration is common in febrile children. However, if correctly hydrated, a febrile child should not be considered as under specific conditions. In hospital, the hydration of febrile infants is under control, but at home, paracetamol is advisable;
- The risk of dehydration due to fever is higher in younger than older children;
- For dehydrated children, the administration of ibuprofen is not necessary and not indicated by the Italian Society of Pediatrics [1].

The inappropriate use of antipyretic drugs for fever or acute mild–moderate pain in children or adolescents can lead to an increased risk of toxicity, especially for specific patients’ profiles.

The expert panels agreed upon identifying specific children’s conditions most amenable to paracetamol or ibuprofen. In conclusion, the opinion of the expert panel was that paracetamol is not only more appropriate for children with specific clinical conditions, but it should also be considered, when required/necessary, the first-line treatment for fever and acute mild–moderate pain.

As an additional suggestion, optimizing the pre- and in-hospital management of fever is desirable. Dehydration, which is common in febrile children [29], should always be addressed.

Furthermore, the maximal daily dose of paracetamol to avoid toxicity is 80–100 mg/kg (the recommended dose is “not to exceed a daily dosage of 60 mg/kg”; the route of administration and the age of the child are important, e.g., in neonates and infants, the dose should be adjusted to 12.5 mg/kg every 6 h if given by the IV route) [1]. Paracetamol (as well as other drugs) poisoning is a common reason of admission in poison centers; therefore, drugs must be kept far out of reach of children.

## 4. Discussion

During the expert opinion consensus process, a particular emphasis was placed on the type of setting. The expert panels agreed on the importance of primary care of patients with fever and pain, using unique patient group identifiers, which are usually symptom-based.

The unmet needs described above, concerning the management of fever and acute mild–moderate pain, can be extended beyond Italy. In particular, the proper use of paracetamol and ibuprofen should be improved everywhere. For example, rectal formulations of paracetamol are still used by parents or prescribed by HCPs regardless of the presence of vomiting or diarrhea [30]. Given the different pattern of side effects, in the experts’ opinion, the proper use of paracetamol and ibuprofen should maximize the benefit and improve the outcome in the short and long terms.

Education about fever management in children can be beneficial not only for parents, but even for HCPs, who can apply heterogeneous approaches [31]. Education may impact the perception and misconception of fever in children (fever phobia) [32]; however, the background education of parents may play a role [4,33].

The current management of pediatric pain calls for a worldwide step forward [2]. A concrete help, in this sense, could in fact be represented by the utilization of standard pain assessment measures [34,35,36,37,38,39]. The assessment and treatment of pain in children and adolescents require constant training to increase clinicians’ awareness. In a retrospective Italian study on pain management in EDs, under-dosing of antalgic drugs was found in 61% (893/1471) of children [7]. This under-dosing was associated with the use of ibuprofen suppositories and lower (<12 kg) or higher body weight (>40 kg) of children [7].

Pain is often under-recognized and under-treated in severely disabled children or adolescents, regardless of the type of disability. This subgroup of patients requires special attention, including on co-morbidities, and validated and specific tools for pain assessment [40,41].

## 5. Conclusions

The experts’ analysis of this study provided a picture of the critical issues of fever and acute mild–moderate pain management in primary care and hospital settings that is similar and generalized in all Italian regions. In light of precision medicine, patients’ profiles can obtain different benefits from paracetamol or ibuprofen according to the different efficacy and safety features of the two drugs.

These expert opinion consensus statements can encourage the dialogue between HCPs and may represent an across-the-board tool to harmonize the routine practice in the different settings. These statements also aim to promote educational activity about fever and acute mild–moderate pain management to enhance the milestones already achieved by Italian pediatricians.

## Figures and Tables

**Table 1:** Pattern of the consensus methods through Workshops 1 and 2. SWOT, strengths, weaknesses, opportunities and threats.

**Workshop 1** **(Ishikawa diagram)**	**Primary care**	Fever	central–northern panel
			central–southern panel
		Acute mild–moderate pain	central–northern panel
			central–southern panel
		Fever plus acute mild–moderate pain in specific patient profiles	central–northern panel
			central–southern panel
	**Hospital settings**	Fever and/or acute mild–moderate pain in general and in specific patient profiles in hospital care settings	central–northern panel
			central–southern panel
**Workshop 2** **(SWOT analysis)**		Paracetamol versus ibuprofen	central–northern panel
			central–southern panel

**Table 2:** The specific comments of the central–northern and central–southern panels that emerged during Workshop 1.

**Primary care**	Fever	Central–northern panel	Lacking distinction between drops and suspension. The administration routes are critical and regard personalization of the dose. Increased use of ibuprofen as a first-line treatment in the territory and EDs due to a perception of ibuprofen having greater efficacy compared to paracetamol. Fever prevention during convulsive crisis not in compliance with the guidelines. Peripherical hospital professionals detect shortcomings in the management of these patients. Use of physical tools, i.e., ice packs and blankets (with no benefit and possibly dangerous). In some areas, this habit is deep-rooted. Difficult management of parents’ anxiety about fever symptoms.
		Central–southern panel	It is necessary to hone the communication modalities for families, i.e., using social media for parents and children and extending the educational activities in schools and on television. Another essential goal is to spread coherent and homogeneous educational messages based on simple concepts (i.e., no physical tools such as ice packs or blankets, and preventive administration of drugs). These messages should be shared between territory and hospital settings. Special attention must be paid to the dosage calculation and improper alternating use of paracetamol and ibuprofen.
	Acute mild–moderate pain	Central–northern panel	To reinforce educational activities, especially for pharmacists. Meetings and training with other HCPs (GPs, orthopedics, otolaryngologists, etc.) can increase awareness about pain in children. Importance of pain prophylaxis.
		Central–southern panel	Pain measurement and knowledge of the tools for pain management are the most critical issues. Notwithstanding the implementation of specific training courses, on-demand therapy persists with a limited approach to pain prophylaxis. This shortcoming is more detectable in departments characterized by a scarce sensibility of pain issues (e.g., orthopedic).
	Fever plus acute mild–moderate pain per specific profiles	Central–northern panel	No comment was stated by central–northern panel in this scenario.
		Central–southern panel	In children with several pathologies, the self-prescription by families must be opposed, and the therapy must be decided by doctors.
**Hospital settings**	Fever or acute mild–moderate pain in general and in specific patient profiles in hospital care settings	Central–northern panel	Prophylaxis for predictable pain, such as that linked to procedures, is needed even for ED-based healthcare professionals.
		Central–southern panel	Excessive use of self-prescribed ibuprofen by the family for fever in children with dehydration and gastroenteritis, possibly explained by an over-prescription of this drug by pediatricians.

ED, emergency department; HCP, healthcare provider; GP, general practitioner.

**Table 3:** The specific comments of the central–northern and central–southern panels that emerged during Workshop 2.

**Central–northern panel**	In patients with varicella, the use of paracetamol is safer than ibuprofen. Parents have a misconception about the superior efficacy and manageability of rectal route. The rectal route has limitations in administering the correct dose, given the variable gut absorption. Parents perceive ibuprofen as more effective than paracetamol. This perception may be due to the use of ibuprofen at the maximum dosage compared to paracetamol, which is often underdosed, especially when administered through the rectal route. Consequently, the rectal route should never be the first-line choice but prescribed only in the presence of vomiting. The gastrolesive side effects of ibuprofen are related only to the subgroup of at-risk children. The risk of ibuprofen is related to the presence of bacterial and primitive diseases, such as tumors, whereby pain and inflammation are epiphenomena.
**Central–southern panel**	Improved awareness of pediatricians and parents about the advantages of paracetamol versus ibuprofen: Possible use in infants and a lower risk of hepatotoxicity. Focus on the rectal route, which should never be the first-line choice, but prescribed only in the presence of vomiting, even if this administration route could be the only way for parents to administer the drug. It is essential to educate parents, since every drug should be administered according to a doctor’s prescription, especially in the territory.

**Table 4:** The statements achieved throughout the consensus process of the current study.

Statement	Central–Northern		Central–Southern	
	Strongly Agree (%)	Agree (%)	Strongly Agree (%)	Agree (%)
1. Recommendations for the use of paracetamol and ibuprofen in the primary care and emergency settings should overlap (* agreement necessary regardless of the setting).	100		75	25
2. The guidelines suggest that the efficacies of paracetamol and ibuprofen are comparable (* in terms of efficacy, a 15 mg/kg dose of paracetamol overlaps a 10 mg/kg dose of ibuprofen).	100		100	
3. Paracetamol showcases a good safety profile when used at the recommended dose of 15 mg/kg 4 times/day maximum (* not to overstep the daily dose of 60 mg/kg; the route of administration and the age of the child are important, e.g., in neonates and infants, the dose should be adjusted to 12.5 mg/kg every 6 h if given by the IV route).	100		100	
4. The use of paracetamol is more appropriate in some specific conditions: Children at-risk of dehydration or dehydrated children and children with varicella, pneumonia, Kawasaki’s disease, or coagulations disorders (* dehydration is frequent condition in infants with fever).	80	20	100	
